# A 2D Electromechanical Model of Human Atrial Tissue Using the Discrete Element Method

**DOI:** 10.1155/2015/854953

**Published:** 2015-10-25

**Authors:** Paul Brocklehurst, Ismail Adeniran, Dongmin Yang, Yong Sheng, Henggui Zhang, Jianqiao Ye

**Affiliations:** ^1^Engineering Department, Lancaster University, Lancaster LA1 4YR, UK; ^2^Biological Physics Group, School of Physics and Astronomy, University of Manchester, Manchester M13 9PL, UK; ^3^School of Civil Engineering, University of Leeds, Leeds LS2 9JT, UK

## Abstract

Cardiac tissue is a syncytium of coupled cells with pronounced intrinsic discrete nature. Previous models of cardiac electromechanics often ignore such discrete properties and treat cardiac tissue as a continuous medium, which has fundamental limitations. In the present study, we introduce a 2D electromechanical model for human atrial tissue based on the discrete element method (DEM). In the model, single-cell dynamics are governed by strongly coupling the electrophysiological model of Courtemanche et al. to the myofilament model of Rice et al. with two-way feedbacks. Each cell is treated as a viscoelastic body, which is physically represented by a clump of nine particles. Cell aggregations are arranged so that the anisotropic nature of cardiac tissue due to fibre orientations can be modelled. Each cell is electrically coupled to neighbouring cells, allowing excitation waves to propagate through the tissue. Cell-to-cell mechanical interactions are modelled using a linear contact bond model in DEM. By coupling cardiac electrophysiology with mechanics via the intracellular Ca^2+^ concentration, the DEM model successfully simulates the conduction of cardiac electrical waves and the tissue's corresponding mechanical contractions. The developed DEM model is numerically stable and provides a powerful method for studying the electromechanical coupling problem in the heart.

## 1. Introduction

Atrial fibrillation (AF) is characterised by rapid and irregular conduction of cardiac electrical excitation waves, impairing the ability of the heart to pump blood via mechanical contraction. AF is the most common cardiac arrhythmia [1], affecting ~1.5% of the UK population, a figure which increases with age (rising to 5% beyond the age of 65 and 10% beyond the age of 75 [2]). It can cause cerebral stroke, incapacitation, and loss of life [3, 4]. Despite this, the underlying processes governing the generation and maintenance of AF are not yet fully understood [5] and current clinical treatments are significantly unsatisfactory [6]. Computational models provide a powerful tool for studying these phenomena and would provide a means of quantitatively predicting the underlying molecular and ionic mechanisms that facilitate the genesis and perpetuation of AF. They also provide a level of control that would not be possible in an experimental setting. Therefore, there is an urgent need to develop biophysically detailed computational models that are capable of capturing various complex mechanisms in the atria, which may then be used to study AF and test potential treatments.

Understanding AF requires thorough analysis of the electrical behaviour of the atria. Highly detailed models of individual cardiac cells have been developed, building on the earlier pioneering work of Hodgkin and Huxley [7] and Noble [8]. These models include the Courtemanche et al. [9] and Nygren et al. [10] models, both of which reproduce the action potential of human atrial myocytes as recorded experimentally. Each model features stiffly nonlinear differential equations simulating the flux of ionic currents to provide the membrane potential of a single cell. Many larger-scale electrophysiological studies exist, which investigate excitation conduction from cell to cell. This presents a challenge to researchers owing to the large computational cost, high speed of the electrical wave, and complex anatomy of the atria [11, 12]. Typically researchers use experimental datasets such as [13] constructing the various atrial regions (pectinate muscle, crista terminalis, Bachmann's bundle, etc.), each of which has different electrical properties. In order to handle such difficulties numerically, a very fine spatial resolution is required in computational models [5, 14, 15]. Full reviews of electrophysiological progress may be found in [16, 17].

The inclusion of mechanical dynamics is vital for a model to address how cardiac arrhythmias or a proposed treatment can affect the mechanical contraction of the heart, especially, since there is strong coupling between electrical and mechanical activities in the atria [18, 19]. Sophisticated models exist at the myocyte/myofilament level [20, 21], capturing the complex force-calcium relationship and sarcomere dynamics. On a larger scale, cardiac tissue may be mechanically idealized as transversely isotropic, hyperelastic, and incompressible [22]. Tissue-/organ-scale models typically use a traditional continuum mechanics approach through materials homogenization, modelling the tension development and deformation by using stress-strain relationships [23–25]. However, a simply idealized continuum approximation of cardiac tissue ignores the pronounced discrete nature of cardiac tissue and cell arrangement and therefore has fundamental limitations.

In this study, we used the discrete element method (DEM) rather than a continuum approach to physically and mechanically represent atrial tissue. DEM belongs to a family of discrete methods originally proposed by Cundall in 1971 in application to the behaviour of discontinuous materials [26]. The method was refined in [27, 28] and describes the motion of circular “particles.” Recently, researchers have shown that particles may be bonded together to accurately model fibre-reinforced materials [29–31].

DEM is therefore well suited in application to atrial tissue, due to the discrete cellular arrangement, the discontinuous tissue's electrophysiological properties, and the complex geometry of the atria. The aforementioned continuum mechanics approaches assume a smooth and homogeneous tissue, neglecting consideration of the discontinuous microstructure and irregular arrangement of cells. In this study, we develop a multiscale DEM model that accurately captures electrical and mechanical processes at both the cell and tissue scales in the atria. We build the groundwork for future model development at the organ scale and show that DEM has the potential to be a powerful approach for representing the anisotropic and inhomogeneous nature of the human heart.

The paper is arranged as follows.  Section 2 provides an overview of DEM theory used by the model, and the atrial single-cell model is presented. The method for electrical propagation is described as well as the coupling with mechanical contraction. Some simulation results are shown in Section 3. A discussion of the method and conclusions are given in Section 4 and some future work is proposed.

## 2. Method

### 2.1. Model for Excitation and Contraction of a Single Cell

The developed biophysically detailed model for the atria considers two physics scales: cellular and tissue. At the cellular level, we simulated the electrical and mechanical behaviour of a single atrial myocyte by coupling two well-known models. For the electrical behaviour, we used the Courtemanche et al. model [9]. Each cell was considered equipotential, with the time derivative of the cell's membrane potential *V* (in mV) given by

(1) $$\begin{array}{c} \frac{dV}{dt}=-\frac{\left(I_{\text{ion}}+I_{\text{st}}\right)}{C_{m}}, \end{array}$$

where *I*_ion_ is the total ionic current (pA), *I*_st_ is an external stimulus (pA), and *C*_*m*_ is the membrane capacitance (pF). Various ionic and pump currents are represented in the model:

(2) Iion=INa+IK1+Ito+IKur+IKr+IKs+ICa,L +Ip,Ca+INaK+INaCa+Ib,Na+Ib,Ca,

where *I*_Na_ and *I*_*b*,Na_ are the fast and background Na^+^ currents, respectively; *I*_K1_, *I*_to_, *I*_Kur_, *I*_Kr_, and *I*_Ks_ are the inward rectifier, transient outward, ultrarapid rectifier, and rapid and slow delayed rectifier K^+^ currents, respectively; *I*_Ca,*L*_, *I*_*p*,Ca_, and *I*_*b*,Ca_ are the L-type, pump, and background Ca^2+^ currents, respectively; *I*_NaK_ is the Na^+^ − K^+^ pump current; and *I*_NaCa_ is the Na^+^ − Ca^2+^ exchanger current. Each ion channel current is modelled by the Huxley-Hodgkin formulation which is fitted to experimental data. For example, the fast sodium current is implemented as

(3) $$\begin{array}{c} I_{\text{Na}}=g_{\text{Na}}m^{3}hj\left(V-E_{\text{Na}}\right), \end{array}$$

where *g*_Na_ is maximal Na^+^ conductance and *E*_Na_ is the equilibrium potential for sodium. The voltage dependency of ion channels is modelled by gating variables *m* (activation), *h* (inactivation), and *j* (slow activation), each governed by

(4) $$\begin{array}{c} \frac{dy}{dt}=\frac{y_{\infty }-y}{\tau_{y}}. \end{array}$$

For any gate variable *y*, *y*_*∞*_ is its steady state value and *τ*_*y*_ its time constant, both of which are functions of *V* that are algebraically defined based on data from atrial cells. Differential equations also exist to keep track of intracellular concentrations of Ca^2+^, Na^+^, and K^+^.

For the mechanical behaviour of the cell, we used the Rice et al. myofilament model [20]. This model defines a system of nonlinear ordinary differential equations to simulate the interaction between force, the intracellular Ca^2+^ handling, and sarcomere length. This is achieved through mathematically modelling the complex process of Ca^2+^ binding to regulatory proteins at cross-bridges. Other realistic elements such as viscoelasticity are included in the model, which is verified against experimental data for cardiac muscle response. The author's implementation defines Integral_force_ as a sum of normalized forces within the cell integrated over time:

(5) Integralforce  =∫−Factive−Fpassive+Fpreload+Fafterloaddt,

with terms representing the contribution of various forces from the model formulation. The sarcomere length SL is then computed by

(6) $$\begin{array}{c} \frac{d\text{SL}}{dt}=\frac{{\text{Integral}}_{\text{force}}+\left({\text{SL}}_{0}-\text{SL}\right)*\text{viscosity}}{\text{mass}}, \end{array}$$

where SL_0_ is initial sarcomere length. Here viscosity and mass are constants which help describe the muscle response [20]. The model is formulated with close attention to experimental data for rat and rabbit myofilaments. Due to lack of experimental data for human contracting cells, we use the rat species modifying parameters of the original paper and a physiological temperature of 37°C. The initial sarcomere length is chosen as SL_0_ = 2.2 *μ*m to reflect possible stretching at end-diastole.

We coupled the Courtemanche et al. and Rice et al. models by a two-way feedback method. The definition for calcium concentration [Ca^2+^]_i_ from the Courtemanche et al. model [9] is modified slightly to give

(7) $$\begin{array}{c} \frac{d{\left[{\text{Ca}}^{2+}\right]}_{\text{i}}}{dt}=\frac{B_{1}}{B_{2}}, \end{array}$$

where *B*_1_ and *B*_2_ are defined by

(8) B1=2INaCa−Ip,Ca−ICaL−Ib,Ca2FaVi +VupIup,leak−Iup+Irel⁡Vrel⁡Vi −2dTropACadt,B2=1+Cmdnmax⁡Km,CmdnCa2+i+Km,Cmdn2,

and other variables are defined as in [9]. Here, we have inserted *d*Trop_*A*_Ca/*dt* as calculated in the Rice et al. model [20]. Values of [Ca^2+^]_i_ are fed into the Rice et al. model, completing the feedback cycle. In this manner, we obtain a system of nonlinear equations which accurately predicts the membrane potential, calcium concentration, and length of a single cell throughout its excitation-contraction cycle. The system consists of nonlinear differential equations for the 32 time-dependent variables. In total, we monitor 182 variables for each cell at each time step, providing diagnostic information on all aspects of single-cell dynamics.

### 2.2. DEM Theory

The mechanical interaction of cells in this study is analysed using DEM theory. The theory in full may be found in the two-part paper [27, 28]. The particular implementation and computational engine utilized in this paper is the Particle Flow Code (PFC) by Itasca Consulting Group Inc. [32].

DEM tracks the dynamic interaction of “particles,” where in this two-dimensional context a particle is defined as a rigid disc of unit thickness occupying a finite space. Each particle's position and velocity are tracked throughout the simulation, which is solved by an explicit time-stepping algorithm. Newton's second law is used to determine the motion of each particle arising from contact/body forces upon it. A force-displacement law is used to update the contact forces arising from the relative motion at each contact [26, 29, 32].

For this two-dimensional study, the degrees of freedom of the particles are the *x*- and *y*-directions and rotation about the *z*-axis. The equation for a single particle's translational motion may be written [31, 32] as follows:

(9) $$\begin{array}{c} F_{i}=m\left({\ddot{x}}_{i}-g_{i}\right), \end{array}$$

where *i* = 1,2, 3 denotes the coordinate directions (*x*, *y*, *z*), *F*_*i*_ is the resultant force (the sum of all externally applied forces acting on the particle), ${\ddot{x}}_{i}$ is the particle acceleration, *m* is the mass of the particle, and *g*_*i*_ is a body force acceleration vector (e.g., gravity). The equation for rotational motion is given by [31, 32]

(10) $$\begin{array}{c} M_{3}=I{\dot{\omega }}_{3}, \end{array}$$

where *M*_3_ is the resultant moment referred to the *z*-axis, *w*_3_ is the rotational velocity about the *z*-axis, and *I* is the rotational inertia of the particle.

**Figure 1:**
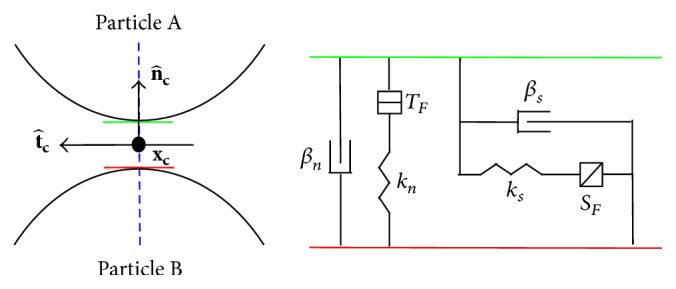
An illustration of the linear contact bond model used in the DEM model, connecting two particles *A* and *B*. Here **x**_**c**_ is the location of the contact plane centred between the two particles, ${\hat{n}}_{c}$ is the normal directed towards particle *B*, and ${\hat{t}}_{c}$ is the tangential direction. *k*_*n*_ and *k*_*s*_ are the normal and shear spring stiffness, *β*_*n*_ and *β*_*s*_ are the dashpot normal and shear critical damping ratios, and *T*_*F*_ and *S*_*F*_ are the tensile and shear strengths of the contact under force.

A solid material may be modelled using DEM by bonding particles together using contact models, and a force-displacement law is solved at the contacts. Two particles may be bonded by a pair of elastic springs with constant normal and shear stiffness (see Figure 1). The bond is retained if the two particles overlap slightly, causing a resistive force, or become slightly separated, causing an attractive force. The springs have specified tensile and shear strength under force, and the contact breaks if these strengths are exceeded. In this study, they are taken high enough for the contacts to persist indefinitely. We use a contact model similar to that described in [33], providing the behaviour of an infinitesimal, linear elastic bonded interface which carries a force. In addition, dashpots are present in the normal and shear direction to provide damping [32].

In order to obtain the spring and dashpot forces acting at a contact, we first define a contact plane between particles *A* and *B* as shown in Figure 1, with location **x**_**c**_ [32]. The contact plane is centred within the interaction area (gap or overlap) of the two particles, with location **x**_**c**_, normal ${\hat{n}}_{c}$ directed from *B* to *A*, and tangential direction ${\hat{t}}_{c}$. The contact model consists of a dashpot and spring in the normal and shear directions. Here *β*_*n*_ and *β*_*s*_ are the dashpot normal and shear critical damping ratios, *k*_*n*_ and *k*_*s*_ are the normal and shear spring stiffness, and *T*_*F*_ and *S*_*F*_ are the tensile and shear strengths of the contact under force. We may thendescribe the relative translational motion of particle surfaces at a contact by

(11) $$\begin{array}{c} \dot{\delta }={\dot{x}}_{c}^{\left(A\right)}-{\dot{x}}_{c}^{\left(B\right)},\\ \dot{\theta }\;=\;w^{\left(A\right)}-w^{\left(B\right)}, \end{array}$$

where ${\dot{x}}_{c}^{\left(N\right)}$ is the translational velocity of particle *N* at the contact location:

(12) $$\begin{array}{c} {\dot{x}}_{c}^{\left(N\right)}={\dot{x}}^{\left(N\right)}+w^{\left(N\right)}\times \left(x_{c}-x^{\left(N\right)}\right). \end{array}$$

Here, ${\dot{x}}^{\left(N\right)}$ and **w** ^(*N*)^ are the respective translational and rotational velocities of particle *N*, and **x** ^(*N*)^ is the location of particle *N* [32]. The relative translation motion $\dot{\delta }$ may be decomposed further into its normal and shear parts:

(13) $$\begin{array}{c} \dot{\delta }={\dot{\delta }}_{n}{\hat{n}}_{c}+{\dot{\delta }}_{s}{\hat{t}}_{c}. \end{array}$$

Now, we denote the total contact force as **F** ^**c**^, with linear and dashpot components:

(14) $$\begin{array}{c} F^{c}=F^{l}+F^{d}, \end{array}$$

and each may be resolved into normal and shear parts:

(15) $$\begin{array}{c} F^{l}=-F_{n}^{l}{\hat{n}}_{c}+F_{s}^{l}{\hat{t}}_{c},\\ F^{d}=-F_{n}^{d}{\hat{n}}_{c}+F_{s}^{d}{\hat{t}}_{c}. \end{array}$$

Finally, the normal contact force is updated at each time step:

(16) $$\begin{array}{c} F_{n}^{l}=k_{n}g_{s}, \end{array}$$

where *g*_*s*_ is the surface gap between the two contacting particles. The shear contact force is updated incrementally, starting from zero and subtracting at each time step:

(17) $$\begin{array}{c} \Delta F_{s}^{l}=-k_{s}{\dot{\delta }}_{s}\Delta t. \end{array}$$

The update of the dashpot normal force is given by

(18) $$\begin{array}{c} F_{n}^{d}=2\beta_{n}\sqrt{m_{c}k_{n}}{\dot{\delta }}_{n}, \end{array}$$

where *m*_*c*_ relates the mass of each particle [32]:

(19) $$\begin{array}{c} m_{c}=\frac{m^{A}m^{B}}{m^{A}+m^{B}}. \end{array}$$

The update of the dashpot shear force is

(20) $$\begin{array}{c} F_{s}^{d}=2\beta_{s}\sqrt{m_{c}k_{s}}{\dot{\delta }}_{s}. \end{array}$$

The contacts transmit only a force and no moment, and slippage does not occur in this model because contacts are always present for any given particle.

Particles in DEM can be grouped to form a “clump” that behaves as one body. The contacts between particles within a clump are skipped to reduce the computational cost, whereas contacts between a clump and any particle external to that clump are treated normally. The particles within a clump may overlap to any extent and will never break apart. Hence, a clump may be used to approximate the shape of any nonspherical body (e.g., cylindrical cells in atrial tissue), by using an arbitrary number of particles.

### 2.3. Applying DEM to Human Atrial Tissue

When choosing how to apply DEM to model human atrial tissue, several considerations must be made. For example, we must first decide how many particles to use in representing one cell (and how to arrange the particles), seeking a balance between realism and computational expense. We must also choose how to introduce force into the system to allow muscle contraction.

DEM gives the option to apply a body force to each particle. For a distribution of particles representing a cell, force could be applied to particles at either end, causing contraction. However, using this approach, it is very difficult to correctly balance the forces within a cell and avoid affecting the neighbouring cells. This leads to unrealistic force accumulation throughout the tissue and an uneven contractile response from each cell. Further, balancing the spring stiffness and particle overlap to satisfy the incompressibility condition of atrial tissue is troublesome. In reality, the protein binding process which results in sarcomere contraction (see, e.g., [34]) causes symmetric cell shortening, which is an independent process for each cell and cells interact mechanically in mostly a supportive context. Therefore, it seems appropriate to instead use one DEM clump to represent one cell. Clumps are rigid with respect to one another and are only able to influence each other's positions, not their size or shape. In order to accurately model contraction of the cell, we manually change the position and size of each clump's particles to match the amount of contraction predicted by the single-cell model in Section 2.1. In this manner, we ensure that each individual cell responds in a mechanically correct way to the electrical stimulus. Also, this approach helps alleviate the computational expense caused by the large number of cells required.

**Figure 2:**
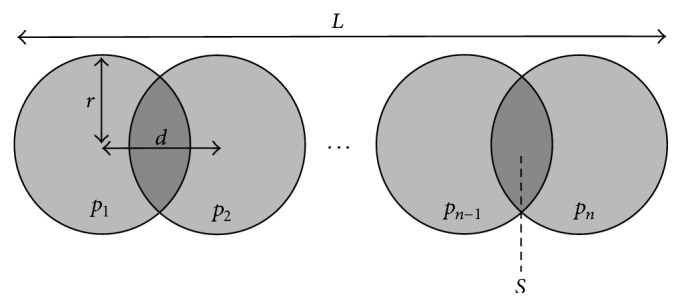
Geometry of one clump/cell in the DEM model. Here, *p*_1_,…*p*_*n*_ are the *n* particles, *r* is their radius, *d* is the distance between particle centres, *L* is the total length of the clump, and *S* is the area of the overlap region between particles.

Atrial cells have a roughly cylindrical shape, and our two-dimensional representation of a cell is a rectangle with initial length 100 *μ*m and width 16 *μ*m as used in the model of Courtemanche et al. [9]. The density of each particle is chosen as *ρ* = 1.053 g/mL, taken from values calculated in [35] for rat myocardial tissue regional densities. In our DEM model, *n* particles are arranged end to end as shown in Figure 2, using a radius of *r* = 8 *μ*m. The number and amount of overlapping of the particles are chosen such that the length of the clump *L* = 100 *μ*m. The amount of overlap is the same for each particle; hence, the equation for the length of the clump is

(21) $$\begin{array}{c} L=\left(n-1\right)d+2r, \end{array}$$

where *d* is the distance from the centre of one particle to the next. In this case we use *n* = 9, giving *d* = 10.5 *μ*m when the cell is at rest. Using simple geometry, the total area *A* of the clump is given by

(22) $$\begin{array}{c} A=n\pi r^{2}-\left(n-1\right)S, \end{array}$$

where the area *S* is

(23) $$\begin{array}{c} S=2r^{2}\arccos\left(\frac{d}{2r}\right)-\frac{d}{2}\sqrt{4r^{2}-d^{2}}. \end{array}$$

At every time step, the single-cell model of Section 2.1 outputs a sarcomere length for each cell. Since each cell consists of sarcomeres arranged end to end, we assume the total cell length is equal to a linear scaling of the sarcomere length. The area *A* is always held constant to satisfy the incompressibility condition of atrial tissue. Solving (21) and (22) simultaneously gives the two unknowns *r* and *d*, and the clump particle radii and positions are then modified to satisfy these new values. In this manner, the cell/clump may contract/expand in length while conserving 2D area.

### 2.4. Cell-Cell Coupling

**Figure 3:**
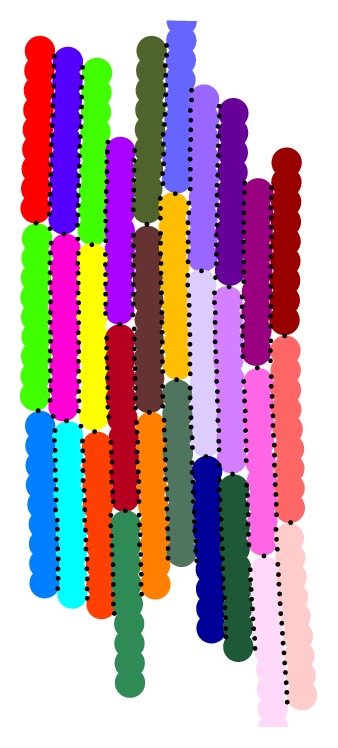
A region of tissue made up of several DEM clumps. Each clump is colored individually, and black circles denote a contact between particles.

Cells need to be coupled both electrically (to allow the electrical excitation waves to propagate through the tissue) and mechanically (to capture the tissue's elastic response to cell contraction). Using DEM, it is simple to construct the mechanical tissue model: clumps are placed in the model where required and aligned with the desired fibre orientation. Mechanical contacts are then formed between clumps/cells and their nearest neighbours. A generous contact detection threshold is set, to ensure a dense arrangement of particles. The DEM distribution is computationally cycled until it reaches a stable equilibrium. Mechanical boundary conditions may be applied to any clump, fixing them in either the *x*- or *y*-directions or both.  Figure 3 displays a DEM model of a region of tissue comprising several cells.

Physically, the DEM contacts in our model represent connective material between individual cells. This includes the cell-binding protein structures desmosomes and other connective tissue. In this study, we seek to capture the qualitative behaviour of these materials, which is primarily to prevent the separation and overlap of cells and facilitate force transmission between cells. Therefore, the DEM parameters are chosen to satisfy the following criteria.

(i) The spring stiffness values *k*_*n*_ and *k*_*s*_ and their ratio are selected such that the mechanical response to cell contraction is prompt and that cells are not separated throughout the contraction process.
(ii) The spring stiffness values are selected high enough that particles resist overlapping, and thus the incompressibility of the tissue is satisfied as closely as possible.
(iii) The spring stiffness and dashpot damping parameters *β*_*n*_ and *β*_*s*_ are selected to minimize elastic oscillation and ensure smooth contraction and expansion of the tissue.

Obtaining the desired behaviour is undertaken through a trial and error process, as the system behaviour is somewhat sensitive to the spring parameters *k*_*n*_ and *k*_*s*_ (and the ratio between them). In general, higher normal contact stiffness *k*_*n*_ causes the above criteria to be satisfied but requires a smaller time step to maintain the stability of the system's solution. However, a critical maximum mechanical time step can be estimated based on parameters [32].  Table 1 shows the full set of DEM parameter values used in this paper. Note that gravity is neglected in this study as the atria are assumed to be supported by the surrounding anatomy.

Recall that clumps are rigid bodies* during* each DEM step. That is, clumps are able to influence each other's position and velocity but* not* each other's size and shape. Instead, the length and width of each clump are manipulated manually prior to each time step, based on the single-cell model output. Then, the DEM calculations give the positional response of the total clump distribution by solving the equations of motion and contact forces. The only force introduced into the system is contact forces between cells, which arises as a result of the contraction/expansion of each cell.

The electrical coupling of cells is performed once the DEM particle distribution is constructed. Two cells are considered electrically coupled whenever a DEM contact exists between them, and we define two such cells as “neighbours.” Equation (1), which governs the membrane potential of each cell, contains a term *I*_st_ which represents an external stimulus. Cells are assumed to stimulate their neighbours, representing electricity flowing from cell to cell. If a cell labelled *H* with membrane potential *V*_*H*_ has *N* neighbours, the external stimulus for cell *H* is given by

(24) $$\begin{array}{c} I_{\text{st}}=D\overset{N}{\underset{i=1}{\sum }}\left(V_{i}-V_{H}\right), \end{array}$$

where *D* is an electrical conductance parameter and *V*_*i*_ is the membrane potential of the neighbour numbered *i*. The conductivity *D* governs the velocity of the electrical wave and has a value of 1000 nS as listed in Table 1. This procedure is repeated for each cell in the tissue, creating one large network of coupled ODEs for the electrical behaviour of all cells in the model.

Physically, electrical propagation from cell to cell is largely due to the presence of gap junctions, a specialized intercellular connection allowing ions and electrical impulses to pass through a regulated gate between cells [36, 37]. Gap junctions are predominantly localized at the cell ends in the fibre direction [38]. However, in our model, the physical connections between cells are the DEM contacts, which naturally are more numerous in the transverse direction than the fibre direction (e.g., see Figure 3). Our approach reflects an attempt to balance this discrepancy by assuming that even if multiple DEM contacts exist between two cells, the electrical flow between them is divided equally amongst these contacts. That is, the number of DEM contacts between two cells does not affect the strength of electrical conduction between them, as long as one contact is present. In Section 3.2, we show that this approach is able to accurately mimic electrical propagation through cells and replicate the anisotropic behaviour of atrial tissue.

**Figure 4:**
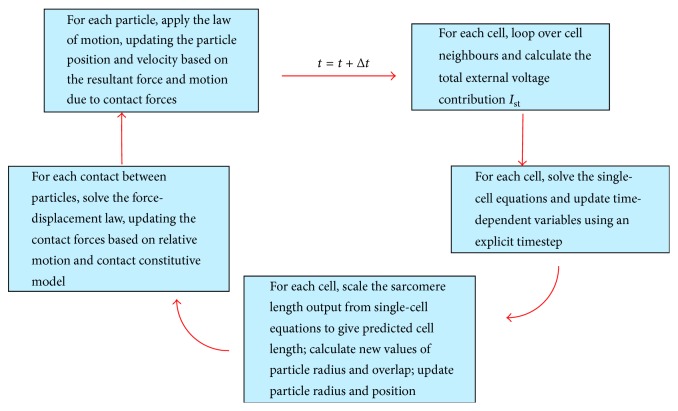
The computational cycle for the full electromechanical model.

Figure 4 shows a schematic of the full electromechanical computational cycle for each time step of the model. The DEM equations are solved explicitly by a centred finite-difference scheme, and a time step of Δ*t* = 0.004 ms is required owing to the relatively stiff contact springs. For the coupled single-cell equations, the same fixed time step of Δ*t* = 0.004 ms is used, to guarantee the stable solution of the coupled system of nonlinear single-cell equations using the explicit Euler method and to handle the high wave speed of the electrical propagation. Each box in Figure 4 must be completed before proceeding to the next; however, they may be computed efficiently in parallel to speed up calculation times. All calculations are performed using an Intel Xeon 3.6 GHz CPU and multithreaded using all 8 threads and double precision. DEM calculations are performed using PFC version 5.0 [32], and all other calculations are performed using a custom C++ library interfacing with PFC. Further computational savings are made by disabling the contact detection phase of DEM during cycling, which is usually present. The particles in this model are densely packed and tightly bonded to their immediate neighbours, meaning no new contacts will be formed throughout the contraction phase.

## 3. Results

### 3.1. Single-Cell Model

**Figure 5:**
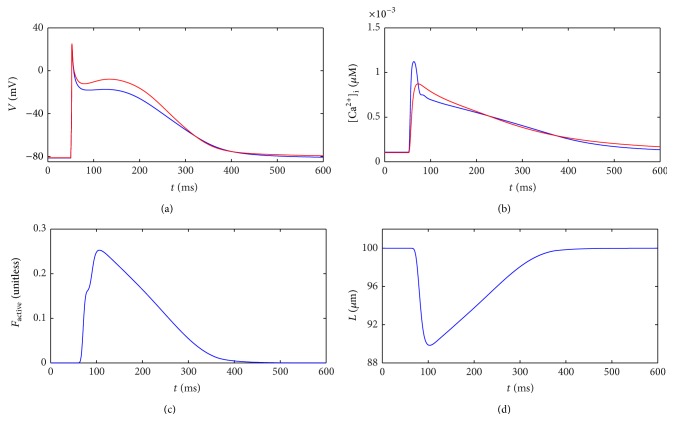
Results from the single-cell model of Section 2.1 (blue curves). The time evolution of several variables is plotted in response to an external stimulus. (a) Membrane potential *V*, (b) intracellular calcium concentration [Ca^2+^]_i_, (c) active force *F*_active_, and (d) cell length *L*. For graphs (a) and (b), the red curves reflect the default Courtemanche model [9].

In this section, we present results from the single-cell model described in Section 2.1. These reflect the electrical and mechanical behaviour of an isolated single cell. At *t* = 50 ms, a 2 nA external stimulus was applied to the cell for a duration of 2 ms. Initial conditions in the cell are obtained by pacing at 1 Hz for 200 beats.  Figure 5 shows the evolution of several variables over time within the cell.

Figure 5(a) shows the action potential, beginning at a resting membrane potential close to −82 mV and reaching 20 mV in response to the external stimulus. The classic spike-and-dome morphology of the Courtemanche et al. model was altered slightly by the mechanical coupling; the dome was less prominent dome and the action potential duration was slightly shorter in the coupled single-cell model (blue) compared to an uncoupled purely electrical model (red) [9]. The abrupt response to the electrical stimulus is evidence of the stiffness inherent in the equations, which must be carefully accounted for numerically.

The Ca^2+^ concentration time course for the coupled model plotted in Figure 5(b) (blue) was affected by the mechanical feedback described in (7)-(8), which caused a sharper and higher peak in Ca^2+^ than in the default electrical model (red) [9]. The influx of Ca^2+^ led to active force development within the cell (Figure 5(c)). This in turn caused a reduction in sarcomere length, which we scaled up to give the expected cell length *L* as shown in Figure 5(d). The cell contracted by around 10% of its original length and was fully contracted 50 ms after receiving a stimulus.

### 3.2. Electrical Conduction

**Figure 6:**
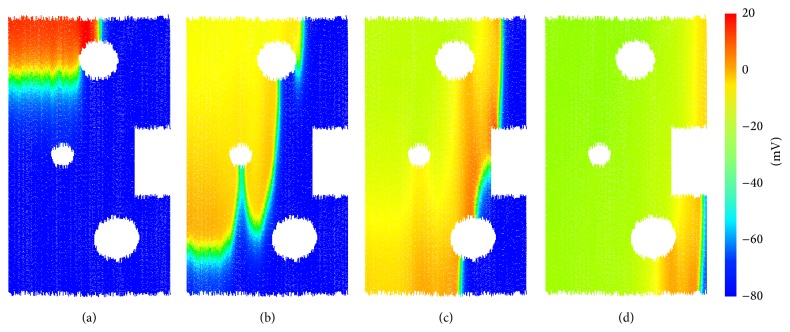
Electrical propagation through a region of connected DEM cells. (a) *t* = 3.92 ms, (b) *t* = 9.12 ms, (c) *t* = 13.52 ms, and (d) *t* = 21.52 ms. Cells are colored according to their membrane potential *V*.

In this section, we demonstrate the method for the electrical coupling of cells, allowing the excitation wave to propagate through the DEM model domain. We construct a rectangular region of tissue, with all cells oriented vertically, such that the fibre direction is in the *y* plane. The tissue consists of 90 k particles (10 k cells), representing a region of tissue which is 5 mm in length and 3.2 mm in width. The top-left corner of cells receives external stimulus of 2 nA at *t* = 1 ms with a duration of 2 ms. The progress of the electrical wave is shown in Figure 6.

Several cavities are opened in the tissue to demonstrate that the method can handle discontinuous tissue geometry. In addition, the anisotropy of atrial tissue conduction is naturally accounted for by the formulation; that is, electrical propagation along fibres is faster than that in the transverse direction. This is due to the larger spatial step in the fibre direction, owing to the clump shape. Here, we have chosen a value for the conduction parameter (*D* = 1000 nS), leading to a conduction velocity of approximately 55 cm/s in the fibre direction, in agreement with experimental measurements [16]. The model exhibits a conduction anisotropy ratio of roughly 6 : 1 between the fibre and transverse directions. The literature estimates for the anisotropy ratio of atrial tissue vary between 4.76 : 1 and 8 : 1 [16] with little experimental data available.

### 3.3. Mechanical Contraction

**Figure 7:**
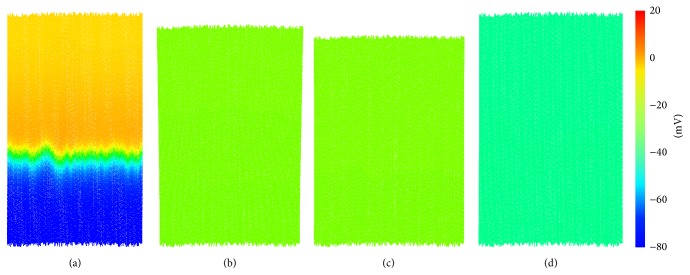
Full electromechanical model for a rectangular region of tissue. (a) *t* = 7.12 ms, (b) *t* = 37.12 ms, (c) *t* = 55.92 ms, and (d) *t* = 225.92 ms.

In this section, we present results from the full electromechanical tissue model described in Section 2.1. A region of tissue 5 mm in length and 3.2 mm in width was constructed with the fibre direction aligned with the *y*-axis, comprised of 10 k cells and 90 k particles. The full simulation took approximately 4 hours to complete 600 ms of simulated time. The computational time is approximately consumed as 50% on DEM calculations, 20% on electrical wave propagation, 20% applying contraction to each cell, and 10% solving the single-cell equations. A boundary condition was applied to the lowermost cells along the *x*-axis, fixing their movement in the *y*-direction. The top portion of tissue received a stimulus of 2 nA at *t* = 1 ms lasting for 2 ms. Several snapshots are presented in Figure 7 as the electrical wave propagates throughout the tissue and contraction occurs.

In Figure 7(a), the rapid electrical wave spreads throughout the cells; by *t* = 37.12 ms (Figure 7(b)), contraction is underway in the cells near the top of the tissue. At *t* = 55.92 ms (Figure 7(c)), the tissue is fully contracted, and the tissue width has expanded to account for its loss in height due to the tissue incompressibility. Gradually, the tissue relaxes to its resting shape (Figure 7(d)).

**Figure 8:**
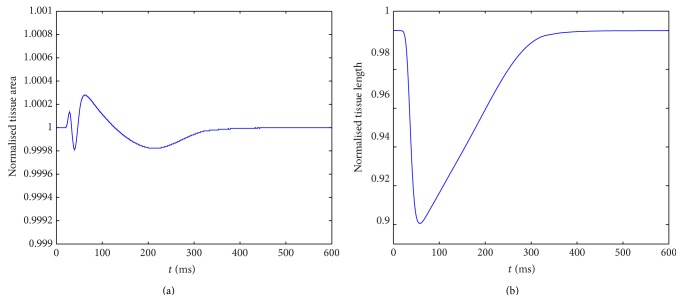
Normalised tissue area (a) and normalised tissue length (b) against time.

To calculate the area of the tissue, we need to account for the area of the physical particles as well as the voids between particles and any particle overlap. We form a polygon with vertices at the centre points of clumps which are on the boundary of the tissue and then calculate the area of the polygon.  Figure 8(a) shows the normalised tissue area (tissue area divided by initial resting area) and Figure 8(b) shows normalised length (distance from the top to bottom through the central fibre divided by the resting length) throughout the contraction.

Figure 8(a) shows that tissue incompressibility is achieved to within acceptable accuracy, with only negligible variation in tissue area throughout the simulation. Recall that the individual cells themselves are exactly incompressible by formulation (see (21)-(22)), but minor changes in tissue area are unavoidable and caused by the clumps and surrounding voids rearranging throughout the contraction. In Figure 8(b), the tissue length curve follows that of each individual cell's length (see Figure 5(d)), showing that cells along a fibre remain unseparated and the stiff springs connecting clumps ensure a prompt response and smooth tissue contraction.

## 4. Discussion

### 4.1. Summary

In this study, we have developed a novel multiscale electromechanical model for the human atria from single cell to tissue level using DEM. Our major contributions are (i) a new cellular model for atrial electromechanical activity that couples the Courtemanche et al. electrophysiology model [9] to the Rice et al. myofilament model [20]. The developed cell model is implemented in DEM by using a clump of nine particles, representing atrial cellular geometry; (ii) a 2D DEM tissue model of atrial tissue was developed with consideration of atrial anisotropic and discrete natures. Using the tissue model, the conduction of cardiac electrical waves and the corresponding mechanical contraction were simulated; and (iii) a numerically stable algorithm was developed to solve the DEM model of atrial tissue.

The developed DEM model presents several advantages over continuum mechanics methods for cardiac tissue modelling, such as the finite element method (FEM). It is well known that cardiac tissue is fundamentally discrete [17], anisotropic, and inhomogeneous in its electrophysiology [39]. Such properties play important roles in ensuring normal electrical wave propagation in the heart and genesis of cardiac arrhythmias [40]. Spatial electrical heterogeneities are also found to be crucial for successful defibrillation [41]. These intrinsic natures of cardiac tissue are more appropriately modelled by DEM than the conventional continuum approaches. Further, the atria are anatomically complex, comprising many regions of different geometrical structures [16], which form a challenge for an accurate reconstruction of these regions and numerical treatment of the nodes by FEM. However, the use of DEM allows any possible arrangement and configuration of individual cells.

The numerical method we presented here was stable and efficient, capable of handling over half a million cells on a single desktop computer. Both the single-cell equations and DEM equations may be solved by explicit numerical methods. Continuum models for electrical propagation frequently use reaction-diffusion type equations [16]: such an approach often requires the iterative solution of a large system of equations [17], slowing down computation times. In addition, the centred finite difference approach to solving the DEM equations used here allows calculation of a critical time step [32], ensuring that the mechanical solution remains stable. Further, in our model there is ample opportunity for parallelism throughout the distinct steps of the computational cycle.

### 4.2. Limitations and Future Work

The present model has several limitations and aspects which may be improved in later model development. First, the single-cell model is not verified against human experimental data, and the Rice et al. [20] model is formulated for nonhuman species. Some authors have attempted to update the Courtemanche and Rice models for use in human models, such as [42, 43]. However, the development of complete electromechanical single-cell models for various regions of the human atria warrants further study as more experimental data becomes available.

The method for electrical conduction between cells needs further improving, though it performs reasonably well. A more sophisticated method could be used, such as tracking the diffusion of electricity through individual particles or direct incorporation of gap junctions. However, these approaches may inhibit the choice of using an equipotential single-cell model. The present method provides a good balance between realism and numerical efficacy.

The focus of the present paper was to introduce the DEM methodology for simulating atrial electromechanics, and there is therefore room for improvement in the future. The DEM bond stiffness and damping parameters can be adapted in different directions to more accurately capture the behaviour of the connective material between cells. A more sophisticated contact model than the linear bond model used here can be developed, possibly including nonlinear effects and viscoelasticity. In addition, to complete the electromechanical coupling, feedback from mechanical contraction can be incorporated into the electrical formulation by introducing a term representing stretch-activated currents [44].

In future, DEM could be used for organ-scale simulations where millions of cells are required. The explicit nature of the numerical methods means the method should scale well to massively parallel systems, using CPU or GPU processors. Construction of an anatomically accurate 3D configuration of cells should be possible. Clumps could be packed into a desired geometry while dynamically modifying their alignment based on experimental data sets defining the atria's fibre orientations. To ensure a dense packing of particles, additional smaller particles could be added, representing connective tissue. In this manner, one could “build” a DEM system of cells to accurately model an atrial region. Though the present paper deals with cells which are aligned in a parallel manner, preliminary tests of our DEM method indicate it is suitable to model abrupt or gradual changes in fibre direction, such as those found in the atria.

### 4.3. Conclusion

We conclude that DEM is a powerful method for modelling electromechanical behaviour in the human atria. The ability to consider discrete cell arrangement means that DEM is well suited to simulate the dynamic behaviour of atrial tissue, which is anisotropic and discontinuous by nature. The multiscale model established here can be used in the future to study the effect of heterogeneity in atrial tissue, which is necessary to fully understand the mechanisms behind AF and other phenomena. The method shows potential to be used for construction of a full 3D model of the human atria, which would provide a valuable computer modelling platform for testing antiarrhythmic drugs and other AF treatments.

## Figures and Tables

**Table 1:** DEM parameter value used in the model.

Parameter	Description	Value
*ρ*	Density of particles	1.053 g/mL
*g*	Acceleration due to gravity	0 m/s^2^
*k*_*n*_	Normal contact stiffness	10 N/m
*k*_*s*_	Shear contact stiffness	0.1 N/m
*β*_*n*_	Normal critical dashpot damping ratio	0.1
*β*_*s*_	Shear critical dashpot damping ratio	0.1
*T*_*F*_	Contact tensile strength	1 × 10^200^
*S*_*F*_	Contact shear strength	1 × 10^200^
*r*	Initial radius of particles	8 *μ*m
*L*	Length of cell	100 *μ*m
*n*	Number of particles per cell	9
*D*	Electrical conductivity	1000 nS
